# p53 Controls Meiotic Prophase Progression and Crossover Formation

**DOI:** 10.3390/ijms23179818

**Published:** 2022-08-29

**Authors:** Marina Marcet-Ortega, Andros Maldonado-Linares, Maria López-Panadés, Ignasi Roig

**Affiliations:** 1 Genome Integrity and Instability Group, Institut de Biotecnologia i Biomedicina, Universitat Autònoma de Barcelona, 08193 Cerdanyola del Vallès, Spain; 2 Cytology and Histology Unit, Department of Cell Biology, Physiology and Immunology, Universitat Autònoma de Barcelona, 08193 Cerdanyola del Vallès, Spain

**Keywords:** p53, meiosis, synaptonemal complex, DSB repair, γH2AX, crossover, MLH1

## Abstract

Meiosis initiates with the formation of double strand breaks (DSBs) throughout the genome. To avoid genomic instability, these DSBs need to be correctly repaired by homologous recombination. Surveillance mechanisms involving the DNA damage response (DDR) pathway ATM-CHK2-p53 can detect the persistence of unrepaired DBSs and activate the recombination-dependent arrest at the pachytene stage. However, a complete understanding of p53 functions under normal physiological conditions remains lacking. Here, we report a detailed analysis of the p53 role during meiotic prophase in mice spermatocytes. We show that the absence of p53 regulates prophase progression by slowing down the pachytene stage when the recombination-dependent arrest occurs. Furthermore, our results show that p53 is necessary for proper crossover (CO) formation and localization. Our study contributes to a deeper understanding of p53 roles during the meiotic prophase.

## 1. Introduction

Meiosis is a specialized cell division that generates haploid gametes from a diploid cell. Haploidization is achieved through two consecutive chromosome segregation events preceded by a single round of DNA replication [1]. Paternal and maternal homologous chromosomes are separated in the first meiotic division, followed by a second meiotic division that pulls apart sister chromatids of each chromosome [2]. Following DNA replication, the first meiotic division starts with an extraordinary prophase, subdivided into four consecutive stages: leptonema, zygonema, pachynema, and diplonema. These stages can be cytologically distinguished by the configuration of the meiosis-specific proteinaceous scaffold that forms along the chromosomes, the synaptonemal complex (SC) [3].

Sporulation-specific protein 11 (SPO11) is a meiosis-specific endonuclease that generates deliberated double-strand breaks (DSBs) throughout the genome at leptonema [4]. A characteristic feature of meiosis is that these DSBs repair through homologous recombination, which uses the homologous chromosome as a template. This process promotes the pairing and synapsis of the homologous chromosomes during the zygonema, reaching full synapsis at the pachynema, followed by desynapsis at diplonema. All this chromosomal choreography is tightly coordinated with the recombination process. In mice, about 10% of the SPO11-generated DSBs will be repaired as crossovers (CO) [5]. The formation of CO implies genetic exchange between the homologs, which increases genetic diversity and provides a physical connection between the homologous chromosomes (chiasmata). These connections ensure correct orientation on the spindle and chromosome segregation to eventually produce balanced haploid gametes [6]. Therefore, defects in CO formation can result in aneuploid gametes, one of the major causes of miscarriages and chromosome abnormality found in humans [7].

Thus, meiotic recombination and homologous chromosome synapsis occur concomitantly in a highly regulated and coordinated manner to successfully obtain viable gametes. For instance, correct repair of the SPO11-induced DSBs becomes a determining process since errors in this may generate genomic instability. Consequently, the meiotic prophase must be subjected to stringent surveillance mechanisms that can detect deleterious events, delay cell cycle to allow their repair, and promote programmed cell death if this is not possible. Remarkably, in many organisms, most known mechanisms that control prophase progression involve the DNA damage response (DDR) proteins ATM and ATR, two conserved serine/threonine kinases that respond to different forms of DNA damage and achieve their functions by phosphorylating a large net of substrates, including CHK2 and p53 family members [8,9,10].

The p53 family genes (*p53*, *p63*, and *p73*) are conserved from invertebrates to mammals and have been found to participate in surveillance mechanisms during meiosis in protecting germline integrity. p53 orthologs in *Drosophila* (Dmp53) and *C. elegans* (CEP-1) are highly expressed in the germ cells. Dmp53 regulates DNA damage-induced apoptosis of germ cells and participates in ovary development [11,12]. Furthermore, Dmp53 is activated in oocytes in response to SPO11-generated DSBs [13]. Similarly, CEP-1 eliminates defective germ cells after irradiation through DNA damage-induced apoptosis [14,15]. In mammals, p53 is highly expressed during spermatogenesis and is activated by SPO11-generated DSBs and irradiated mouse spermatocytes [13,16,17,18,19]. p53-dependent apoptosis was first observed on *Arf* mutant spermatocytes, which display increased levels of γH2AX at pachynema [20]. Taking advantage of defective DNA repair in the hypomorphic *Trip13* mutants, it was later described that in mouse spermatocytes, both p53 and TAp63 block prophase progression at an early pachytene stage in the presence of persistent unrepaired DSBs [21]. Similarly, p53 (and TAp63 to a lesser extent) also regulates the elimination of oocytes with persistent unrepaired DSBs and synapsis defects [22,23]. The TAp63 isoforms undergo phosphorylation and are highly expressed in mouse ovaries and testis [24,25]. In the absence of *p63*, meiocytes appear to progress normally [26]. However, p63 is necessary to eliminate irradiated dictyotene-arrested oocytes, protecting them from genomic instability [27,28]. TAp73 is also highly expressed in oocytes, and TAp73-deficient females presented more oocytes with spindle abnormalities and altered folliculogenesis [29]. Male mice lacking *p73* presented impaired spermatogonial proliferation with increased DNA damage and apoptosis on the seminiferous tubules, resulting in a reduction of spermatocytes and spermatids [29,30].

Altogether, all this evidence highlights the critical role of p53 family members in protecting the germline from DNA damage and maintaining genomic stability. The first studies that analyzed *p53* null mice reported normal development and proper fertility [31,32]. Nonetheless, others described that partial impairment of p53 expression was associated with the presence of testicular multinucleated “giant-cells”, attributed to spermatocytes being unable to repair DSBs [33]. However, a complete understanding of how recombination and synapsis progress in the absence of p53 remains unknown. Therefore, we performed a comprehensive analysis of the *p53* mutant meiotic prophase phenotype in mouse spermatocytes. Our findings revealed that p53 is necessary to control meiotic prophase progression, as expected from its known role in the recombination-dependent arrest. Surprisingly, we found that p53 is also required to regulate CO formation in spermatocytes. This finding suggests the role of the DDR signaling pathway in CO control.

## 2. Results

### 2.1. Testis Size in p53^−/−^ Mutant Mice Is Not Altered

**Figure 1:**
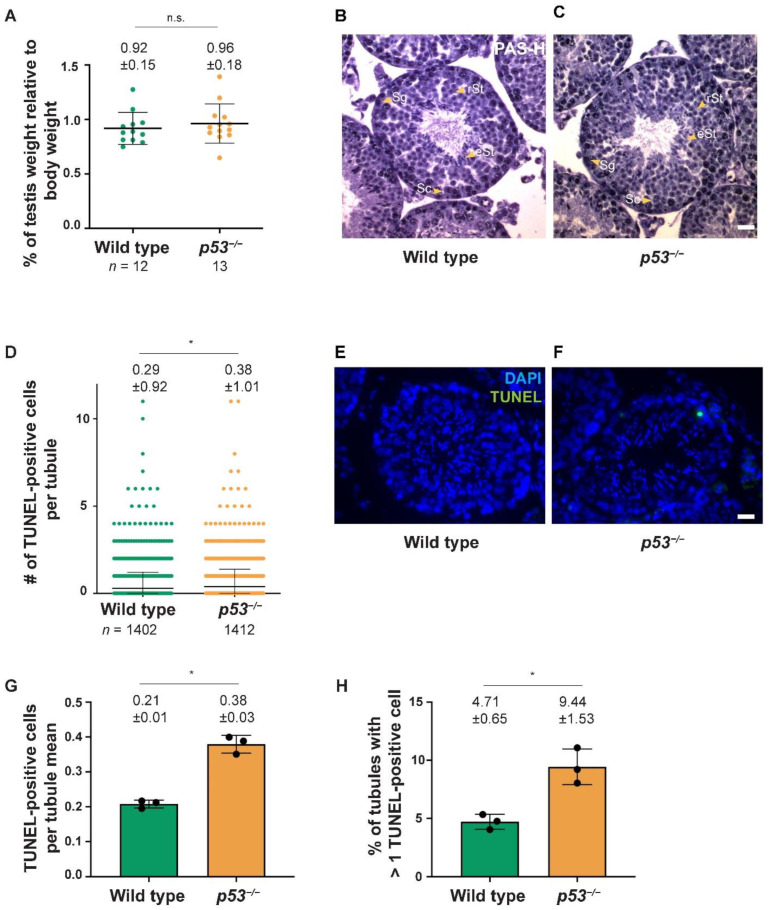
The absence of p53 does not grossly alter spermatogenesis. (**A**) Graph showing normalized testes weight (testes weight divided by body weight) of wild type and *p53^−/−^* testes. Horizontal lines represent means ± SD. Means (±SD) are indicated above the graph; (**B**,**C**) Representative tubule sections from adult testes of the indicated genotypes stained with PAS-hematoxylin. Wild-type and *p53^−/−^* seminiferous tubules contain spermatogonia (Sg), spermatocytes (Sc), round spermatids (rSt), and elongated spermatids (eSt). The scale bar in (**C**) represents 20 µm and applies to all panels; (**D**) Quantification of the number of TUNEL-positive cells per tubule of the indicated genotypes. Horizontal lines represent the mean ± SD, which is indicated over the graph (mean ± SD), and (*n*) shows the number of tubules counted per genotype; (**E**,**F**) Representative tubules are shown from testis sections of a wild-type and *p53^−/−^* testis stained with TUNEL to detect apoptotic spermatocytes (green) and DAPI (blue). Scale bars in (**C**,**F**) represent 20 µm and apply to all panels; (**G**) Bar graph shows average TUNEL-positive cells for wild-type (*N* = 3) and *p53^−/−^* testes (*N* = 3). Bars represent mean ± SD; (**H**) Bar graph represents percentage of tubules with more than one TUNEL-positive cell, for wild-type (*N* = 3) and *p53^−/−^* testes (*N* = 3). In panels (**A**,**D**,**G**,**H**), the results of *t*-tests are indicated as (n.s.) not significant and (*) *p* < 0.01; Bars represent mean ± SD.

Testicular size is a good indicator of spermatogenesis progression, as most of the mutations that compromise meiosis reduce testis weight. Therefore, first of all, we analyzed testes size relative to the body weight. *p53^−/−^* mouse testes weight (Mean ± SD= 0.96 ± 0.15) was comparable to their wild-type littermates (0.92 ± 0.18, *p* = 0.51 *t*-test; Figure 1A). This was an expected result, as it was previously described that *p53^−/−^* mice were fully fertile [32].

To better understand how meiosis proceeds in the absence of p53, we examined histological sections of *p53* mutant mouse testes. Germ cells going through spermatogenesis develop within the seminiferous tubules arranged in circular layers. Pre-meiotic cells (spermatogonia) are located at the periphery of the tubule, and as they progress through meiosis (spermatocytes), they advance toward the lumen until becoming post-meiotic cells (round and elongated spermatids) [34]. Histological cross-sections from *p53^−/−^* testes showed that, as in the wild-type mice (Figure 1B), all spermatogenic cell types were present in seminiferous tubules of *p53* mutant mice (Figure 1C). We did not find any “giant cells” in our mutant sections, which is consistent with the fact that this degenerative phenotype depends on the genetic background of the mice and is not present in C57BL/6 and 129/Sv mixed background mice [33], like the one we have in our colony.

Increased apoptosis in seminiferous tubules can also be an indicator of impaired meiosis. Therefore, we performed a TUNEL assay on histological sections to determine if apoptosis levels are altered in *p53* mutants (Figure 1D–F). We observed a statistically significant increase in apoptotic cells per tubule in *p53^−/−^* testes sections (Mean ± SD = 0.38 ± 1.01) compared to the wild-type (0.29 ± 0.92, *p* = 0.009, *t*-test; Figure 1D). The increase was also observed when comparing the average TUNEL-positive cells per tubule for each individual mouse (*N* = 3 wild-type mice and *N* = 3 *p53^−/−^* mice) (Mean ± SD = 0.21 ± 0.01 for wild-type versus 0.38 ± 0.03 in *p53^−/−^*, *p* = 0.0004, *t*-test; Figure 1G). By analyzing the percentage of tubules with more than one apoptotic cell, we found that *p53* mutants present more tubules with > 1 TUNEL-positive cells (*N* = 3, Mean ± SD = 4.71 ± 0.65) than wild-type mice (*N* = 3, Mean ± SD = 9.44 ± 1.53, *p* = 0.0079, *t*-test; Figure 1H). Nonetheless, we think this increased apoptosis might not be biologically relevant, as we and others have reported *p53^−/−^* mice are fully fertile [32]. Furthermore, we have not seen any sign of spermatogenesis arrest in *p53^−/−^* mice (see above), which suggests this increase in apoptotic cells might not be relevant for mice fertility. Thus, testis size and histological analysis showed that spermatogenesis is not appreciably compromised in the absence of *p53*.

### 2.2. Prophase Progression Is Slightly Accelerated in the Absence of p53

Because p53 is implicated in cell cycle control as a checkpoint protein in somatic cells [35] and it was found that its absence accelerated the initiation of meiotic prophase in females [36], we exhaustively studied meiotic progression in *p53^−/−^* spermatocytes. The first wave of spermatogenesis is semi-synchronous and begins around six days post-partum (dpp). The pachytene stage appears at approximately 12–14 dpp, and the first spermatocytes that initiate the diplotene stage appear around 17–18 dpp. After this first meiotic wave, male meiosis becomes asynchronous, and thus, spermatocytes at all meiotic prophase stages can be found in all adult testis samples.

**Figure 2:**
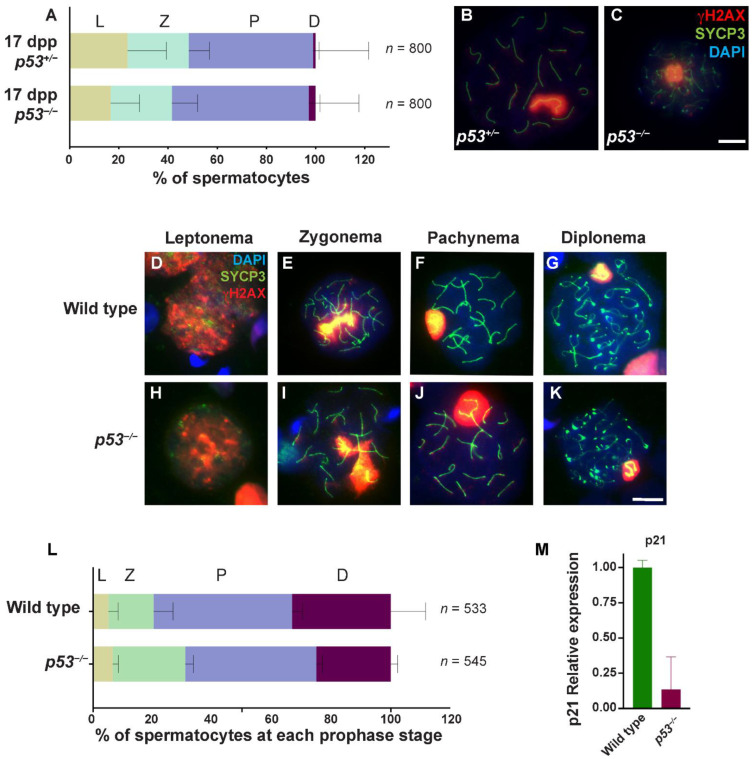
The absence of p53 alters meiotic prophase progression in spermatocytes. (**A**) Quantification of the percentage of spermatocytes at each prophase stage (leptonema (L), zygonema (Z), pachynema (P), and diplonema (D)) at 17 dpp in *p53^−/−^* and *p53^+/−^* mice. (*n*) shows the number of spermatocytes counted per genotype. Bars represent mean ± SD for *N* = 3 *p53^+/−^* mice and *N* = 3 *p53^−/−^* mice; (**B**) Representative *p53^+/−^* pachytene spermatocyte; (**C**) *p53^−/−^* diplotene spermatocytes stained against the axial element protein SYCP3 (green), γH2AX (red), and DAPI (blue); (**D**–**K**) Spread chromosomes from representative spermatocytes of *p53^−/−^* and wild-type mice from leptonema to diplonema stage, stained against SYCP3 protein (green), γH2AX (red), and DAPI (blue); (**L**) Quantification of the percentage of spermatocytes at each prophase stage in *p53^−/−^* and wild-type adult mice. (*n*) shows the number of spermatocytes counted per genotype. Bars represent mean ± SD. Scale bar in (**C**,**K**) represent 10 µm and apply to all panels; (**M**) Graph shows relative RNA expression of p21 in wild-type (green) and *p53^−/−^* (red) mouse testis by RT-qPCR. Bars represent mean ± SD.

To evaluate whether p53 monitors meiotic progression, we studied the chronology of the first wave of meiosis in juvenile mice. We performed immunofluorescence in 17 dpp spermatocyte spreads, marking the axial element of the synaptonemal complex (SYCP3) to follow synapsis progression and γH2AX to observe DSBs and sex body formation. We observed a similar prophase progression pattern in wild-type and *p53* mutant mice. We observed proper synapsis of the homologous chromosomes and prophase progression in adult *p53^−/−^* spermatocytes. Interestingly, when we compared the percentage of spermatocytes that reached the diplotene stage at 17 dpp, we found a significant increase in the percentage of cells at diplonema in *p53^−/−^* mice (2.75%) in comparison with control heterozygotes (1.00%, *p* = 0.0152 Fisher’s exact test; Figure 2A and Figure S1A and Table S1). We also observed a substantial reduction of *p53^−/−^* spermatocytes at leptonema (16.75%) compared to control heterozygotes (23.63%, *p* = 0.0008 Fisher’s exact test, Figure 2A and Figure S1A and Table S1). Therefore, these results suggested that the meiotic prophase is accelerated in the absence of p53, possibly because p53 controls meiotic prophase progression [21].

Next, we studied adult testis and corroborated that synapsis progression in *p53* mutants behaved like the wild type (Figure 2D–K). At the onset of the prophase, the axial elements began to form in each homolog, and the γH2AX signal covered most chromatin marking the recently formed DSBs. As these DSBs were repaired at the zygotene stage, the γH2AX signal decreased, and the homologous chromosomes were progressively paired and synapsed. At the pachytene stage, we observed full synapsis of the homologous chromosomes, and γH2AX was mainly confined to the sex body (X and Y chromosomes). At diplonema, homologous chromosomes desynapsed and decondensed, and the signal at the sex body became fainter.

We also analyzed the proportion of spermatocytes at each prophase stage. We observed that meiotic prophase progression was altered in *p53* mutant mice (*p* = 0.00047, G test). Specifically, there was a statistically significant increase in the number of zygotene stage spermatocytes in *p53^−/−^* mice (24.42%) compared with the wild-type (15.23%; *p* = 0.0027, Fisher’s exact test; Figure 2L and Figure S1B and Table S2). Additionally, the percentage of spermatocytes at diplonema was significantly reduced in *p53* mutant mice (24.96 vs. 33.13%; *p* = 0.0393, Fisher’s exact test; Figure 2L and Figure S1B and Table S2).

In somatic cells, p53 can regulate G1/S and G2/M cell-cycle checkpoints by preventing cell division progression. Upon activation, p53 upregulates several effectors. Among them, p21 induces the G1/S checkpoint and is responsible for maintaining the G2/M arrest. p21 expression was detected in wild-type mouse testis by Western blot and by immunohistochemical staining p21 was found in pachytene spermatocytes. Thus, we investigated whether p21 expression was altered in *p53* mutant mice. To do so, we extracted RNA from adult control and mutant mouse testis and performed an RT-qPCR with specific primers against p21. We found *p53* mutant mice had, on average, a reduction of 99.5% of p21 expression as compared to wild-type (Figure 2M). These data suggest that p21 might be involved in the regulation of the progression along the meiotic prophase in mice.

These alterations suggest that p53 controls meiotic progression in adult spermatocytes. Interestingly, the effect of the lack of p53 in juvenile and adult mice might be the opposite. While in juveniles absence of p53 leads to a faster meiotic prophase progression, in adults, it leads to a slower progression, evidenced by an accumulation of zygotene stage cells.

Altogether, these results indicate that, although p53 controls meiotic prophase progression, it is dispensable to complete meiotic prophase correctly, as expected from a putative checkpoint protein [21].

### 2.3. CO Formation and Location Are Altered in p53 Absence

**Figure 3:**
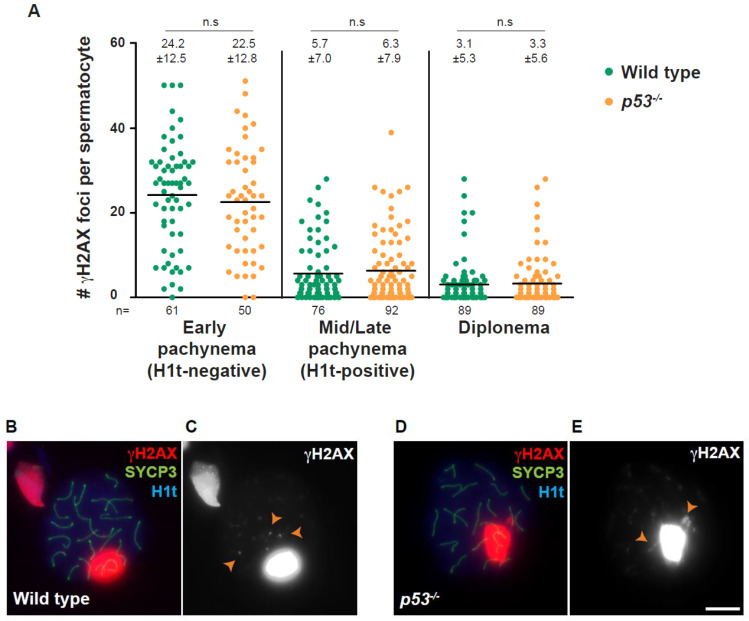
The absence of p53 does not affect DSB repair efficiency. (**A**) Quantification of the number of γH2AX foci per spermatocyte found at each indicated stage in wild-type and *p53^−/−^* mice. The large, bright blobs of γH2AX are the sex bodies, and the smaller foci reflect unrepaired DSBs (orange arrowheads). Horizontal lines represent means ± SD. Means (±SD) are indicated above the graph. (n.s.) indicates *t*-test is not significant. The number of cells counted (*n*) is indicated below the graph; (**B**–**E**) Representative pachytene spermatocytes are shown from wild-type and *p53^−/−^* mice, stained against γH2AX (red), SYCP3 (green), and H1t (blue). The scale bar in (**E**) represents 10 µm and applies to all panels.

Next, we studied the completion of meiotic recombination in the absence of *p53.* We used γH2AX as a DSB marker (Figure 3B–E) and examined the number of γH2AX foci present at pachynema and diplonema. We found similar numbers of γH2AX foci at early pachynema between *p53* mutant and wild-type cells (*p* = 0.49, *t*-test; Figure 3A). We used H1t protein as a mid/late pachynema marker. At this stage, the number of γH2AX foci presented no significant differences between *p53^−/−^* and wild-type spermatocytes (*p* = 0.56, *t*-test; Figure 3A–E). Finally, we obtained an equivalent number of γH2AX foci in *p53* mutant and wild-type spermatocytes at diplonema (*p* = 0.77, *t*-test; Figure 3A). Therefore, these results suggest that *p53* absence does not grossly alter DSB repair completion dynamics.

**Figure 4:**
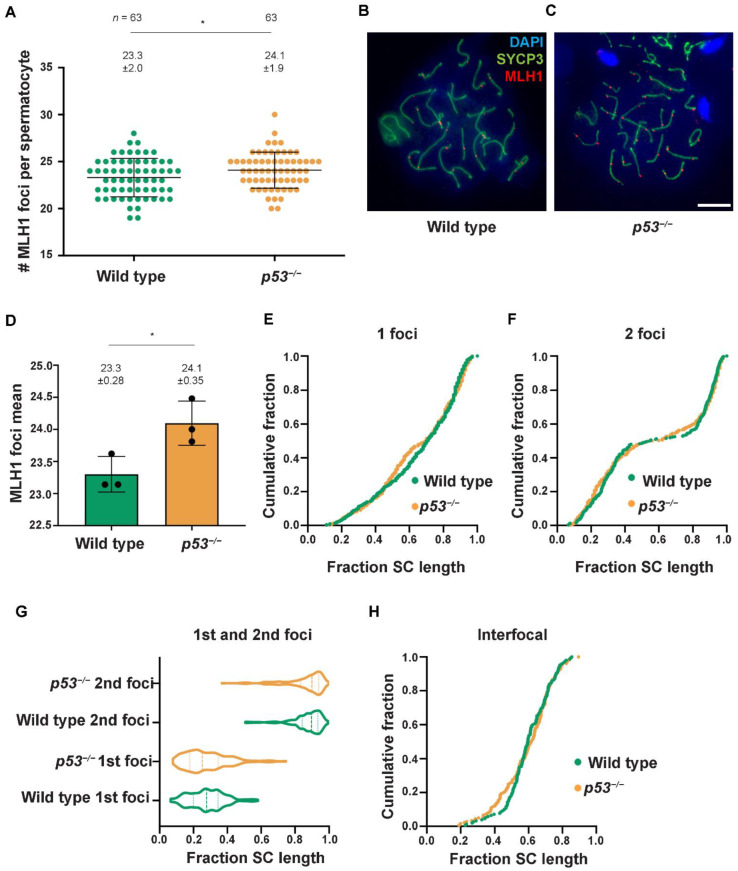
The absence of p53 alters CO formation. (**A**) Quantification of the number of MLH1 foci per spermatocyte at the pachytene stage. Horizontal lines represent means ± SD. Means (±SD) are indicated above the plot, and (*) indicates statistically significant *p* < 0.05 for the Mann-Whitney test. The number of cells counted per genotype (*n*) is also indicated; (**B**,**C**) Representative pachytene spermatocytes are shown from wild-type and *p53^−/−^* mice, stained against MLH1 (red), SYCP3 (green), and DAPI (blue). The scale bar in (**C**) represents 10 µm and applies to all panels; (**D**) Bar graph shows MLH1 foci mean for each individual mouse (*N* = 3). Means (±SD) are indicated above the plot, (*) indicates statistically significant *p* < 0.05 for *t*-test; (**E**) Graph shows MLH1 foci distribution as a cumulative fraction along the SC length for bivalents with a single MLH1. The X-axis represents the relative length from the centromeric end (left) to the distal telomere (right); (**F**) Graph plots the cumulative fraction of MLH1 foci for bivalents with two MLH1 foci; (**G**) Violin plot represents the distribution of the first and second foci along the relative SC length in chromosomes displaying two MLH1 foci; (**H**) The cumulative fraction of inter-focus distances is shown.

Since most DBSs do not repair as crossovers (COs) [37], we also characterized CO formation in the absence of *p53*. About two hundred DSBs are generated at leptonema by the SPO11 protein during mouse meiosis, but only 10% of these recombination sites become COs. COs provide a physical linkage between the homologous chromosomes, ensuring accurate segregation during the first meiotic division. CO formation is tightly regulated, and each pair of homologous chromosomes gets at least one CO. To study CO formation, we used the mismatch repair protein MLH1, which localizes at CO sites at pachynema (Figure 4A–C). We observed a significant increase of MLH1 foci in *p53^−/−^* spermatocytes compared to wild-type (*p* = 0.036, Mann-Whitney test; Figure 4A). The MLH1 foci increase in *p53^−/−^* spermatocytes is also observed when analyzing the means for each individual mouse (*N* = 3, *p* = 0.0356, *t*-test; Figure 4D).

In mice, the DDR tightly regulates CO position to ensure proper segregation of homologous chromosomes at metaphase I [38]. Thus, we wondered if the absence of p53 could affect where COs were placed on the chromosome. We measured the relative position of MLH1 foci in each bivalent and compared the distribution between *p53^−/−^* and control spermatocytes. To analyze the cytological distribution of MLH1 foci, we first compared bivalents containing one MLH1 focus in wild-type and *p53^−/−^* spermatocytes. In wild-type cells, most MLH1 foci tended to accumulate at the distal telomeric end of the bivalent, as previously reported [39,40]. However, *p53^−/−^* cells had a higher proportion of interstitially placed foci. While only 9% of wild-type MLH1 foci accumulated around 0.45–0.55 SC relative length, 17% of *p53^−/−^* foci were placed in this region (*p* = 0.0008, Fisher’s exact test, Figure 4E). This increased presence of MLH1 foci in the mid-section of the chromosome was also apparent in the *p53^−/−^* bivalents with two MLH1 foci. In wild-type cells, the two MLH1 foci were well separated and only overlapped in 2.5% of the pairs of foci (*n* = 155). In contrast, *p53^−/−^* spermatocytes distribution of the first and second MLH1 foci overlapped in 21.0% of the pairs (*n* = 181; *p* < 0.0001; Fisher’s exact test, Figure 4F,G). This translated into a reduced cytological interference observed in the mutant spermatocytes, as measured by the adjustment of the interfocal distances to the gamma distribution (wild-type γ = 18.8 vs. *p53^−/−^* γ = 12.7, Figure 4H).

These results show that p53 is dispensable to form COs but is required to form the correct number of COs and properly place them in the genome, at least at a cytological level. These data emphasize the role of the DDR in regulating the CO location in mammals.

## 3. Discussion

Meiotic prophase progression requires the coordination of several processes, such as recombination, synapsis, and chromosome segregation. Germ cells undergo dramatic reorganizations, starting with induced programmed DSBs throughout the genome. These SPO11-generated DSBs are repaired through homologous recombination, which promotes proper homologous pairing and synapsis and eventually gives rise to CO formation (the physical linkage that ensures correct chromosome segregation). If deleterious events, such as unrepaired DSBs or asynapsis, occur, meiotic surveillance mechanisms delay prophase progression and eliminate defective meiocytes by activating programmed cell death. Several studies have highlighted the role of the DNA damage response pathway in the meiotic checkpoint network in several organisms [9,10].

We had previously described that the DDR pathway participates in the meiotic checkpoint, where the MRE11 complex-ATM-CHK2-p53/TAp63 pathway activates the recombination-dependent arrest in mouse spermatocytes [21,41]. Therefore, in this present study, we wanted to evaluate meiotic prophase progression in the absence of *p53*. Previous reports support a p53 role in mouse meiosis, as p53 is physiologically activated in response to SPO11-induced DSBs [13], and *Arf^−/−^* spermatocytes experience p53-dependent apoptosis [20]. Similar results have been observed in other organisms, suggesting a conserved role of p53 in responding to DNA damage in the germinal line. For instance, *Drosophila* transgenic GFP-p53 expression was observed in female oocytes in a Spo11-dependent manner (*Drosophila* males do not present meiotic recombination) [13]. *C. elegans* CEP-1 is required to activate irradiation-induced apoptosis of germ cells (although not in somatic cells), but did not activate cell cycle arrest [14]. This observation was similar to what was found in *Drosophila* cells, where p53 homolog activates DNA damage-induced apoptosis but not cell cycle arrest [42]. The reason why CEP-1/p53 only induces germ cell apoptosis might be related to *C. elegans* somatic cells having a limited number of possible cell divisions. Therefore, while somatic apoptosis would be extremely deleterious for the animal, damaged germ cells that were not eliminated by apoptosis would give rise to defective offspring. This logic supports the first role of p53 as a germline protector and later evolving as a tumor suppressor in somatic cells [14].

Although some studies have tried to evaluate a possible role of p53 in spermatogenesis, a clear role of p53 beyond the activation of the recombination-dependent arrest remains ambiguous. Mutant mice lacking p53 are viable, fertile, and develop normally. Nevertheless, they are already highly prone to develop tumors at the age of six months [31,32]. *p53* null mice present reduced litter size due to embryonic lethally of a fraction of *p53* null embryos. These lost embryos are primarily females, showing neural tube closure defects and exencephaly [43,44,45]. The interest in analyzing the p53 role during the meiotic prophase also came from the observation that p53 is expressed in primary spermatocytes, and the highest levels are observed at pachynema [16,17,18]. Additionally, p53 expression increased after irradiation in spermatocytes and spermatogonia, suggesting a role in DNA damage response in germ cells [18,19]. Moreover, multinucleated degenerative giant cells were detected in testis sections, presumably caused by impaired meiotic divisions. These giant cells were only observed in some mouse strains, like the 129/Sv strain, but not in C57BL/6 × 129/Sv mixed genetic background [33]. Our results are consistent with this observation since we did not observe any multinucleated cells in our samples (also coming from C57BL/6 × 129/Sv mixed background).

Some reports have tried to specifically answer if p53 participates in DNA repair or recombination in mouse spermatogenesis. For instance, one study found an increased number of γH2AX foci in *p53^−/−^* pachytene spermatocytes and an absence of sex body in some pachynema cells (in a CBA background). They also reported that Caspase-3 was slightly increased in *p53^−/−^* mouse seminiferous tubules, accounting for spermatogonia or secondary spermatocytes at stage XII [46]. In another work, recombination frequency analysis using four polymorphic microsatellite markers revealed no significant differences between p53 mutants or wild-type offspring [45]. We also observed a slight increase in apoptotic cells, and we similarly observed that the TUNEL-positive cells were mainly somatic cells. These results suggest that p53 might be involved in the DSB repair of testis somatic cells and that the observed apoptosis is p53-independent.

We did not observe any synapsis defects in our p53 mutants. Regarding meiotic prophase progression, we could only observe that in the absence of *p53*, there was an acceleration of prophase progression in the first meiotic wave, suggesting a role for p53 in checkpoint activation. Interestingly, similar results were found for p53 mutant oocytes [36]. These results suggest a role of p53 in checkpoint activation even where no external irradiation or additional mutations are causing high or persistent DSBs repair intermediates. These findings are in consonance with our previous work describing the p53 role in activating the recombination-dependent arrest, where spermatocytes with elevated unrepaired DSBs are arrested at the early pachytene stage by p53 and TAp63 [21].

Regarding meiotic recombination, our analysis of DSB repair using γH2AX foci as recombination markers did not reveal any differences between the wild-type and *p53* mutants, suggesting that the absence of p53 does not affect DNA repair in mouse spermatocytes. We suggest that we obtained different results than the previously reported [46], which may account for the usage of different mouse genetic backgrounds.

We also analyzed CO formation in *p53* mutants. To our surprise, COs were significantly higher in *p53^−/−^* spermatocytes than in controls. This result suggests that p53 regulates the outcome of meiotic recombination. However, since the increase only represents one extra CO per spermatocyte, we speculate that this difference might not have a relevant biological effect in *p53^−/−^* recombination frequency, as reported before and corroborated by the fact that *p53* mutants are fertile [45]. There are several ways in which the absence of p53 could increase the number of COs, such as affecting synaptonemal complex length or CO interference. Alternatively, it should be further studied if p53 can interact with MLH1 and alter its function.

Our analysis of the location of COs in the bivalents suggests that the absence of p53 influences CO positioning and weakens CO interference, thus resulting in an increased number of COs per spermatocyte. This is not the first member of the DDR signaling pathway involved in CO control since previous studies have evidenced that ATM regulates proper CO formation in mice [38]. Interestingly, a possible role of p53 in CO control has been suggested in other organisms. *C. elegans* CEP-1 (p53 homolog) was found to be necessary for proper X chromosome segregation during meiosis [14], and female flies lacking *p53,* although being fertile, presented a reduced crossover rate [13]. More recently, it has been proposed that CEP-1, in collaboration with Him-5, participate in the DSB repair pathway choice [47]. Thus, the fact that there seems to be a conserved role of p53 in modulating the recombination outcome supports our results. Overall, the observed participation of p53 in regulating meiotic progression and CO formation suggests a possible role of p53 in coordinating these events.

## 4. Materials and Methods

### 4.1. Mutant Mice

Mice carrying *p53* mutations were previously generated and described elsewhere [32,48,49,50,51,52]. These lines were purchased from Jackson Laboratories (Bar Harbor, ME, USA) and maintained in a C57Bl/6 × 129/Sv mixed background. All experiments were performed using at least two animals (unless mentioned in the text) and compared with control littermates when possible or from animals of closely related parents. For adult mice, testis from 2 to 4 months old mice were collected and processed.

DNA was extracted from the mouse tails for PCR analysis for genotyping. The following conditions were used: predenaturation at 94 °C for 2 min, then 25 cycles of denaturation at 94 °C for 45 s, annealing at 60 °C for 1 min, and extension at 72 °C for 1 min. *Trp53* deletion was detected with primers: 5′-ACAGCGTGGTGGTACCTTAT, 5′-TCCTCGTGCTTTACGGTATC, and 5′-TATACTCAGAGCCGGCCT, which amplify fragments of 375 bp for the wild-type allele or 525 bp for the mutant. Experiments performed in this study complied with EU regulations and were approved by the Ethics Committee of the UAB and the Catalan Government.

### 4.2. Histology

Testes were fixed either with Bouin’s solution or 4% paraformaldehyde for histology, embedded in paraffin, and sectioned into 6-µm sections. For histological staging analysis [34], sections fixed with Bouin’s solution were stained with Periodic Acid-Schiff (PAS) and hematoxylin. Histological sections fixed with paraformaldehyde were used for apoptosis analysis. The In situ Cell Death Detection kit (Roche Diagnostics, Basel, Switzerland) was performed following the manufacturer’s instructions to detect apoptotic cells.

### 4.3. Spermatocyte Spreads and Immunofluorescence

Surface-spread nuclei spermatocyte preparations were made from fresh mouse testes. Seminiferous tubules were released into 1x PBS to be minced with the scalpel. The cell suspension was transferred into 10 mL of PBS and homogenized. One milliliter of cell suspension was transferred into 1.5 mL tubes, centrifuged, and the supernatant was removed. Pellets were resuspended into 40 µL of 0.1 M sucrose solution at 37 °C and incubated at room temperature for 5–10 min. Then 20 µL of cell suspension was added to Superfrost slides with 65 µL of cold fixative solution (1% paraformaldehyde, 0.1% Triton X-100 and protease inhibitor cocktail (Roche Diagnostics, Basel, Switzerland) in MilliQ-water, pH = 9.2). Slides were placed into a humid chamber for 2 h at room temperature. Then the chamber was opened to let slides air dry for 30–60 min. Slides were rinsed with 0.4% Photo-Flo solution (Kodak, Rochester, NY, USA) for 2 min, air dried, and stored at −80 °C.

Spermatocyte preparations were immunostained using the following protocol. Slides were blocked with PTBG (0.2% BSA, 0.2% gelatin, 0.05% Tween-20 in PBS) for 10 min in agitation. Primary antibodies used were mouse anti-SYCP3 (Abcam, Cambridge, United Kingdom, 1:400), rabbit anti-SYCP3 (Abcam, Cambridge, United Kingdom, 1:400), mouse anti-γH2AX (Millipore, Burlington, MA, USA, 1:400), guinea-pig anti-H1t (kind gift from M. A. Handel, Jackson Laboratory, Bar Harbor, ME, USA, 1:500), mouse anti-MLH1 (G168-15) (BD Biosciences, Franklin Lakes, NJ, USA, 1:50). Secondary antibodies were all raised in goat and conjugated with FITC, Cy3, or Cy5 (Jackson Immunoresearch Europe, West Grove, PA, USA). Slides were mounted with Vectashield mounting medium (Vector, Newark, CA, USA) containing DAPI. Images were captured in a Zeiss Axioskop microscope (Zeiss, Oberkochen, Germany) using ProgRes C10 camera using ProgRes Pro 2.7.7 software (Jenoptik, Jena, Germany) and processed with Photoshop (Adobe, San Jose, CA, USA).

Statistical analysis GraphPad Prism 9 software (Graphpad software Inc., San Diego, CA, USA) was used for Student’s t and Mann-Whitney tests and or GraphPad QuickCalcs online resource (http://www.graphpad.com/quickcalcs/ (accessed on 31 March 2016)).

### 4.4. RNA Extraction and RT-qPCR

RNA was extracted from testis using the RNeasy Plus mini kit from Qiagen (Hilden, Germany) following the manufacturer’s instructions. A cDNA library was produced from 2 μg of total RNA with the iScript Advanced cDNA Synthesis (Bio-rad, Hercules, CA, USA). RT-PCR was performed in a CFX96 Real-Time PCR Detection System thermocycler (Bio-Rad, Hercules, CA, USA) and analyzed with Bio-Rad CFX Manager software (Bio-Rad, Hercules, CA, USA). *p21* and the housekeeping gene *Gapdh* primers were obtained from Biorad (Bio-Rad, Hercules, CA, USA). All samples were run in triplicate, and non-template controls were included in the analysis.

## Data Availability

Not applicable.

## Supplementary Materials

The supporting information can be downloaded at: https://www.mdpi.com/article/10.3390/ijms23179818/s1.
