# Supplementary material for: p53 Controls Meiotic Prophase Progression and Crossover Formation
Source: Int J Mol Sci. 2022 Aug 29;23(17):9818. doi: 10.3390/ijms23179818 (PMC9456223; doi:10.3390/ijms23179818)
Supplement: Supplementary file 1 [file ijms-23-09818-s001.zip › ijms-1720658-Figure S1.pdf]

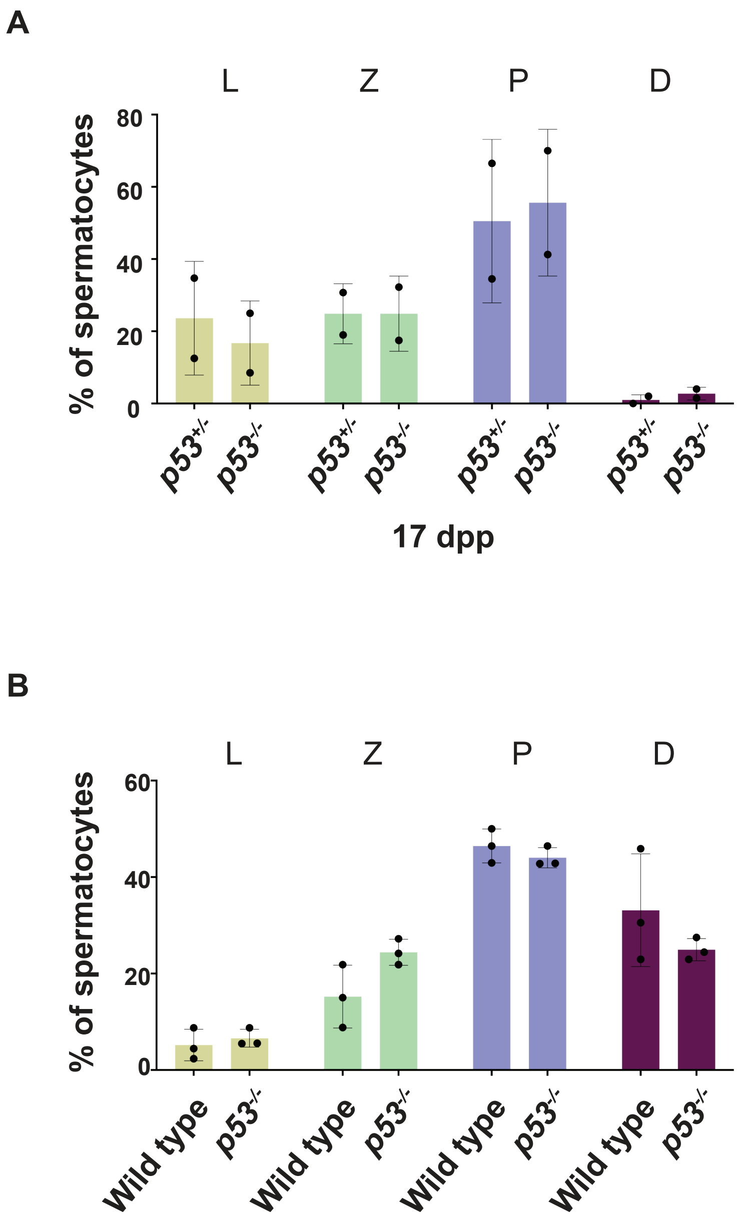

Supplemental Figure S1. Meiotic prophase stages progression in p53 deficient spermatocytes.
(A) Bar graph shows percentage of spermatocytes at each prophase stage (leptonema (L), zygonema (Z), pachynema (P), and diplonema (D) at 17 dpp in p53+/- (N=2) and p53-/- (N=2) mice. Bars represent mean ± SD and black dots correspond to each individual mouse. (B) Bar graph represents percentage of spermatocytes at each prophase stage (leptonema (L), zygonema (Z), pachynema (P), and diplonema (D) in wild-type (N=3) and p53-/- (N=3) adult mice. Bars represent mean ± SD and black dots correspond to one mouse analyzed.
