# Supplementary material for: p53 Controls Meiotic Prophase Progression and Crossover Formation
Source: Int J Mol Sci. 2022 Aug 29;23(17):9818. doi: 10.3390/ijms23179818 (PMC9456223; doi:10.3390/ijms23179818)
Supplement: Supplementary file 1 [file ijms-23-09818-s001.zip › MarcetOrtega_Table_S1.pdf]

Table S1. Distribution of spermatocytes along the meiotic prophase in juvenile mice

**17dpp juvenile**	**% Leptonema**	**% Zygonema**	**% Pachynema**	**% Diplonema**	Cells analyzed	Mice analyzed
p53 $^{+/-}$ (mean±SD)	23.63 ±15.73	24.88 ±8.31	50.50 ±22.63	1.00 ±1.41	800	2
p53 $^{-/-}$ (mean±SD)	16.75 ±11.67*	24.88 ±10.43	55.63 ±20.33*	2.75 ±1.77*	800	2

***** Significantly different from *p53+/-,* P < 0.05 Fisher's exact test.
