# Supplementary material for: p53 Controls Meiotic Prophase Progression and Crossover Formation
Source: Int J Mol Sci. 2022 Aug 29;23(17):9818. doi: 10.3390/ijms23179818 (PMC9456223; doi:10.3390/ijms23179818)
Supplement: Supplementary file 1 [file ijms-23-09818-s001.zip › MarcetOrtega_Table_S2.pdf]

Table S2. Distribution of spermatocytes along the meiotic prophase in adult mice

**Adult mice**	**% Leptonema**	**% Zygonema**	**% Pachynema**	**% Diplonema**	Cells analyzed	Mice analyzed
**Wild type** (mean ±SD)	5.18 ±3.26	15.23 ±6.52	46.46 ±3.53	33.13 ±11.68	533	3
p53 $^{-/-}$ (mean ±SD)	6.60 ±1.86	24.42 ±2.69*	44.03 ±2.10	24.96 ±2.30*	545	3

* Significantly different from wild type, P < 0.05 Fisher's exact test.
